# Human papillomavirus testing in primary screening for the detection of high-grade cervical lesions: a study of 7932 women

**DOI:** 10.1054/bjoc.2001.1845

**Published:** 2001-06

**Authors:** C Clavel, M Masure, J-P Bory, I Putaud, C Mangeonjean, M Lorenzato, P Nazeyrollas, R Gabriel, C Quereux, P Birembaut

**Affiliations:** Laboratoire Pol Bouin, C.H.U. de Reims 45, rue Cognacq-Jay, 51100 Reims, France; Department of Obstetrics and Gynecology, C.H.U. de Reims, 51100 Reims, France; Department of Cardiology and Department of Statistics, C.H.U. de Reims, 51100 France

**Keywords:** HPV, cervical cancer, screening

## Abstract

High-risk human papillomaviruses (HR-HPV) are the necessary cause of cervical carcinomas. To determine whether HPR-HPV DNA detection in primary routine screening could represent a sensitive and reliable technique for the detection of high-grade squamous intraepithelial lesions (HGSIL), laboratory analysis using 2 cytologic techniques (conventional and liquid-based), HPV testing with Hybrid Capture II assay (HC-II), followed by colposcopic examination of women with abnormal cervical finding and/or persistent HR-HPV infection, was conducted in 7932 women who had routine cervical examination. The sensitivity of HPV testing for detecting a histologically proven HGSIL was 100%, higher than that of conventional (68.1%) and liquid-based (87.8%) cytology. The low specificities of 85.6% and 87.3% of HPV testing slightly increased to 88.4% and 90.1% if HPV testing was reserved for woman >30 years old. The quantitative approach provided by the HC-II assay for the assessment of the viral load was not reliable for predicting HGSIL in normal smears. HR-HPV testing could be proposed in primary screening in association with cytology. With conventional cytology it significantly improves the detection of HGSIL. With the use of the same cervical scrape for HPV testing and liquid-based cytology, HR-HPV testing would allow to select positive samples treated in a second time for cytology which gives a good specificity. © 2001 Cancer Research Campaign http://www.bjcancer.com


*British Journal of Cancer* (2001) **89**(12), 1616–1623
© 2001 Cancer Research Campaign
doi: 10.1054/ bjoc.2001.1845, available online at http://www.idealibrary.com on IDEAL®

### Human papillomavirus testing in primary screening for the detection of high-grade cervical lesions: a study of 7932 women

**C Clavel[1], M Masure[1], J-P Bory[2], I Putaud[1], C Mangeonjean[1], M Lorenzato[1], P Nazeyrollas[3], R Gabriel[2], C Quereux[2] and P Birembaut[1]**

[1]Laboratoire Pol Bouin, C.H.U. de Reims, 45, rue Cognacq-Jay, 51100 Reims, France; [2]Department of Obstetrics and Gynecology, C.H.U. de Reims, 51100 Reims, France; [3]Department of Cardiology and Department of Statistics, C.H.U. de Reims, 51100, France

**Summary** High-risk human papillomaviruses (HR-HPV) are the necessary cause of cervical carcinomas. To determine whether HPR-HPV DNA detection in primary routine screening could represent a sensitive and reliable technique for the detection of high-grade squamous intraepithelial lesions (HGSIL), laboratory analysis using 2 cytologic techniques (conventional and liquid-based), HPV testing with Hybrid Capture II assay (HC-II), followed by colposcopic examination of women with abnormal cervical finding and/or persistent HR-HPV infection, was conducted in 7932 women who had routine cervical examination. The sensitivity of HPV testing for detecting a histologically proven HGSIL was 100%, higher than that of conventional (68.1%) and liquid-based (87.8%) cytology. The low specificities of 85.6% and 87.3% of HPV testing slightly increased to 88.4% and 90.1% if HPV testing was reserved for woman >30 years old. The quantitative approach provided by the HC-II assay for the assessment of the viral load was not reliable for predicting HGSIL in normal smears. HR-HPV testing could be proposed in primary screening in association with cytology. With conventional cytology it significantly improves the detection of HGSIL. With the use of the same cervical scrape for HPV testing and liquid-based cytology, HR-HPV testing would allow to select positive samples treated in a second time for cytology which gives a good specificity. © 2001 Cancer Research Campaign http://www.bjcancer.com

**Keywords**: HPV; cervical cancer; screening

It is now well established that oncogenic (high-risk) human papillomaviruses (HPV) are a causal factor in the development of cervical intraepithelial and invasive neoplasias (Lorincz et al, 1992; Zur Hausen, 1994; Bosch et al, 1995; Walboomers et al, 1999). Infections with high-risk HPV (HR-HPV) are associated with a relative risk of between 8 and 11 for the development of squamous intraepithelial lesions (SIL) and essentially low-grade SIL (LGSIL) containing HR-HPV progress to high-grade SIL (HGSIL) (Koutsky et al, 1992; Gaarenstroom et al, 1994). Because of this, there is an increasing interest in using HPV DNA detection either alone or in addition to classic cytologic examination, essentially as a method for triaging women with a cytologic diagnosis of atypical squamous cells of undetermined significance (ASCUS) in their cervical smears (Cox et al, 1995; Wright et al, 1998; Manos et al, 1999) and now for primary cervical screening (Cuzick et al, 1995, 1999; Clavel et al, 1998a, 1999; Meijer et al, 1998; Kuhn et al, 2000; Ratnam et al, 2000; Schiffman et al, 2000). Nevertheless, the low sensitivity of HPV detection in previous studies has led some authors to consider that HPV testing does not appear to be of value for identifying women with abnormal smears who could be safely followed up with cytology alone (Kaufman and Adam, 1999). Moreover, the low positive predictive value and the cost-effectiveness of HPV DNA testing have to be taken in consideration for primary screening. However, HPV DNA testing in cervical scrapes offers a diagnostic assay without the sampling problems and subjectivity of cytology, but such an approach needs a specific, sensitive, reliable and easy to perform method.

*Received 25 January 2001*
*Revised 7 March 2001*
*Accepted 20 March 2001*

*Correspondence to:* C Clavel

5 years ago, a commercial HPV detection test, Hybrid Capture-I (HC-I), followed by second-generation test Hybrid Capture II (HC-II) was introduced (Lorincz, 1996). HC-II is a non-radioactive, reproducible, relatively rapid, liquid hybridization assay in microtitres designed to detect 18 HPV types divided into high-risk (types 16, 18, 31, 33, 35, 39, 45, 51, 52, 56, 58, 59, and 68) and low-risk (types 6, 11, 42, 43 and 44) groups. The sensitivity of this assay is quite similar to that of Polymerase Chain Reaction (PCR) (Clavel et al, 1998b; Peyton et al, 1998; Riethmuller et al, 1999).

Nevertheless current screening remains based on the cytopathologic classification of cervical smears, according to the Bethesda system, leading to colposcopy and histological sampling. Women in wealthier countries are largely protected via the Papanicolaou test. However, this test is not perfect and false-negative rates of 5 to 50% have been reported (Schneider et al, 1996; Cuzick et al, 1999). Thus, in the last few years, significant technical advances have raised the possibility of improving conventional cytology. Particularly, liquid-based cytology has improved sampling, fixation, staining and background, ensuring a more representative sample with a dramatic improvement in sensitivity (Papillo et al, 1998; Sherman et al, 1998; Weintraub and Morabia, 2000).

Recently, Cuzick et al (2000) have emphasized the urgent need to undertake a large trial of HPV testing in conjunction with other new technologies including liquid-based cytology. Here we report our experience on HPV testing in primary screening of 7932 women, for the detection of HGSIL. For that, we compared the results of HPV

testing to the results of conventional and liquid-based cytology. Because most LGSIL regresses spontaneously, our primary endpoint was the histological diagnosis of HGSIL at the biopsy.

### MATERIAL AND METHODS

### Study population

A total of 7932 women with a median age of 34 years (range 15 to 76 years) were recruited for the study between August 1997 and February 2001. This population was restricted to women who underwent their biennal or triennial routine screening in the Department of Obstetrics and Gynecology of the CHU of Reims. We excluded subjects on the basis of a recent cytologic abnormality and/or an untreated cervical lesion in the past 2 years, pregnant women and patients with AIDS. All women were informed of the aim of the study and gave their consent.

### Cytologic diagnosis

At the first gynaecologic examination, in 2281 women, 2 samples were taken: first, a cytologic smear with an Ayre's spatula, for classical cytology, then one scrape for the HC-II test with a Cervexbrush (Medscan, Uppsala, Sweden). These samples were suspended in 1 ml of specimen transport medium for HPV testing (Digene, Silver Spring, MD). In another way, 5651 women had only one cervical scrape with a Cervexbrush at the first entry. Samples were prepared for liquid-based cytology with the ThinPrep technique (Cytyc Corporation, Marlborough, Mass) and 4 ml of the sample were used for HPV testing. In total, considering the first samples and the follow-up of women, we collected 10 101 cervical smears and scrapes. Smears were classified according to the Bethesda system for reporting cervical or vaginal cytological diagnosis. We selected women with adequate smears including metaplastic and/or endocervical cells according to the criteria of Bethesda, which represented 92% of our total smears. However, even if the smear was not considered as adequate, we also included women with smears evocative of lesions. In another way, 4.8% of the samples treated for liquid-based cytology could not be used for HPV testing and have been excluded. The cytotechnicians and pathologists involved in the study were not informed about the results of the HPV testing. All smears showing cytological abnormalities and biopsy specimens were examined by the same 2 independent pathologists, without knowledge of cytology results for the biopsies examination. The results were compared and if the first 2 diagnoses disagreed, a third pathologist reviewed the case with no knowledge of preceding diagnoses. Consensus diagnoses were determined by two-thirds majority when possible and remaining discrepancies resolved by conference review. Patients with HGSIL were systematically treated by loop electrosurgical excision procedure (LEEP). Data from these LEEP specimens were included in the disease definitions.

### Colposcopic referral

In our protocol (Table 1), all the women presenting cytological abnormalities evocative of cervical lesions (from ASCUS to HGSIL) were systematically recalled for colposcopy in the next few weeks, at an interval ranging from 14 to 160 days (mean 95 days) after entry examination. Punch biopsy specimens were taken from the areas colposcopically suspicious for CIN. All the women with smears within normal limits but presenting a HR-HPV infection were also systematically recalled 6 months later for a new cytological examination and HR-HPV testing followed by colposcopy if a lesion and/or a persistent HR-HPV infection was detected. Punch biopsy specimens were taken from the areas colposcopically suspicious for CIN. The women with a second HR-HPV test positive without any detectable lesions were recalled 6 to 12 months later for a third control with cytological examination and HR-HPV testing and the same indications as above for colposcopy and biopsy. Women with regressive HR-HPV infection were also recalled for colposcopy 12 months later. By contrast, women with initial normal smears and without any HR-HPV infection were followed with a classical biennal or triennal cervical screening with a new cytological examination and HR-HPV testing at the second control. Some of these women had also randomly a colposcopic examination. There were not particular selection criteria for colposcopy. The primary endpoint of our study was the detection of a histologically proven HGSIL at the biopsy and/or on the LEEP specimen.

### HPV testing

When conventional cytology was performed, specimens for HPV DNA testing were suspended in 1 ml of ViraPap/Viratype transport medium (Digene, Silver Spring, MD) and stored at –20°C until further processing. When samples were used for liquid based cytology, 4 ml of the sample were centrifuged and the cell pellet was resuspended in 200 µl of phosphate-buffered saline for HPV testing. HPV DNA detection was performed by the commercially available HC-II System (Digene). All scrapes were analysed for the presence of HR-HPV types 16, 18, 31, 33, 35, 39, 45, 51, 52, 56, 58, 59 and 68. This enzyme-linked immunosorbent assay is based on a sandwich hybridization followed by a nonradioactive alkaline phosphatase reaction with chemoluminescence in microplates. The chosen positive threshold of this test was 1.0 pg $ml^{-1}$ of HPV DNA.

Samples were classified as positive for HR-HPV DNA if the relative light unit (RLU) reading obtained from the luminometer was equal to or greater than the mean of the 3 positive control values supplied by the HC-II kit. As some authors have reported that increasing HPV DNA levels of HR-HPV types were the principal predictors of CIN (Cox et al, 1995), we used as proposed, the ratio RLU/positive controls values to quantify HR-HPV DNA in our samples. Moreover, we added other positive controls such as SiHa cell lines (1 to 2 copies of HPV type 16 per cell) to check the reproducibility of the HC-II sensitivity.

### Statistical methods

The statistical methods used were mostly descriptive. Sensitivity, specificity, positive and negative predictive values were determined by comparing the results of each test to the gold standard of histology. A few high-grade lesions may have been missed if they were negative on both tests and thus women were not referred for colposcopy. 95% confidence interval for these values were assessed using either binomial or normal distribution, according to the data. Moreover, differences between HR-HPV detection and cytologic diagnosis values were compared using Fisher exact test or Chi-2 statistics as adequate, with a $P$ value set to 5%.

### RESULTS

At their first examination, in our population of 7932 women, 1214 (15.3%) presented a HR-HPV infection.

© 2001 Cancer Research Campaign

**Table 1** Protocol for the follow-up of the women

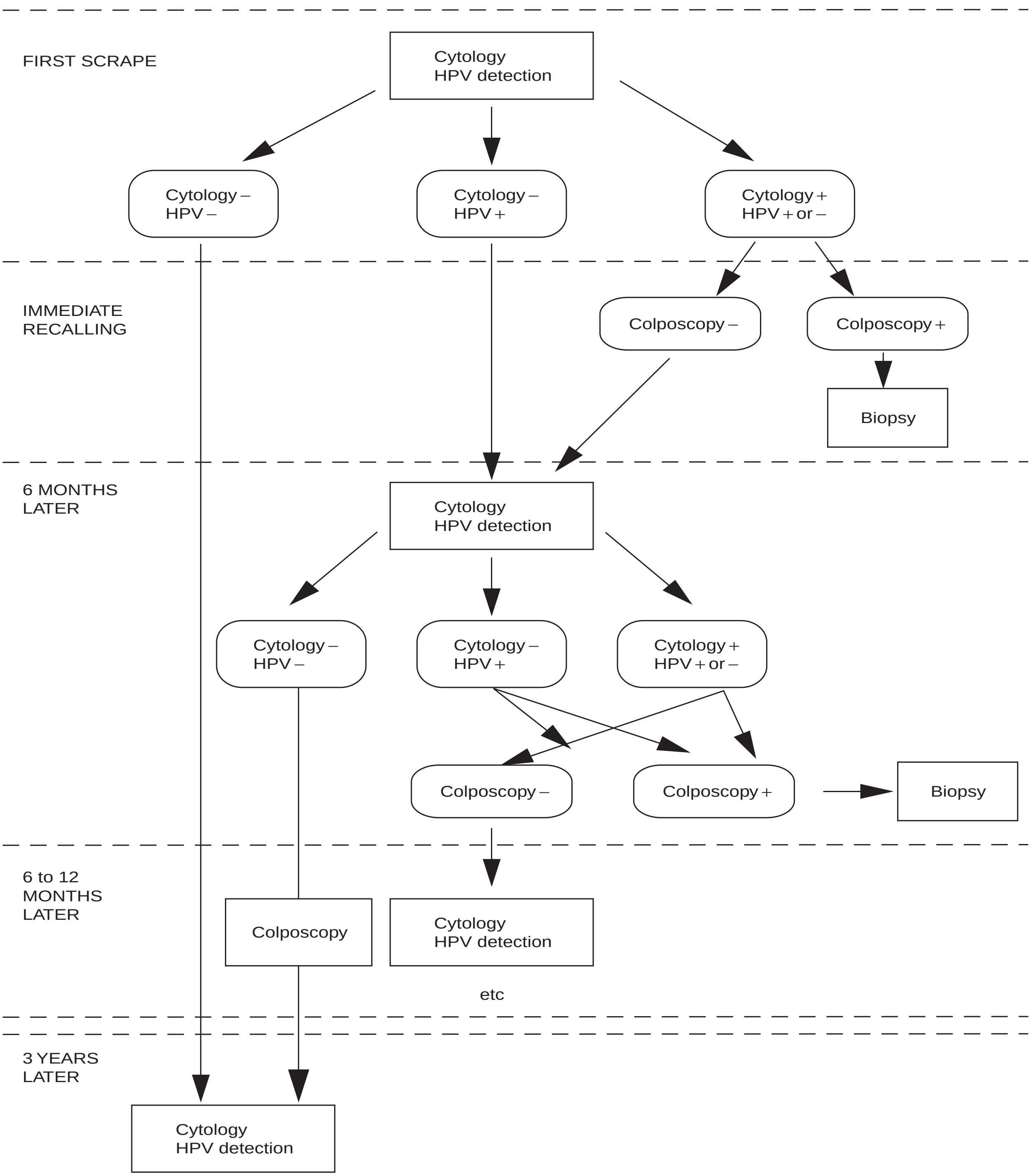

Table 2 represents the prevalence of HR-HPV infection observed in our 7932 women according to their age, at the first examination. There was a peak of infection in the third decade (23.6% of women) with a progressive decrease after 30 years.

### HPV detection and cytological and histological diagnoses

These results are summarized in Table 3.

The prevalence of HR-HPV infection was significantly related to the severity of the cytologically detected lesions ($P < 0.001$).

*© 2001 Cancer Research Campaign*

**Table 2** Prevalence of high-risk HPV infections according to age at the first examination

| Age | Women | High-risk HPV |
|---|---|---|
| < 20 | 418 (5.3%) | 84 (20.1%) |
| 21–30 | 1843 (23.2%) | 435 (23.6%) |
| 31–40 | 2076 (26.2%) | 289 (13.9%) |
| 41–50 | 1925 (24.3%) | 235 (12.2%) |
| 51–60 | 1014 (12.8%) | 110 (10.8%) |
| > 60 | 656 (8.3%) | 61 (9.3%) |
| Total | 7932 | 1214 (15.3%) |

25 women had a smear evocative of HGSIL at the first examination with conventional cytology with a HR-HPV infection in all of them. The diagnosis of HGSIL was histologically confirmed in 21 of them. 82 women had a smear evocative of HGSIL with liquid-based cytology, with a HR-HPV positive test in 79 (96.3%) of them. The diagnosis of HGSIL was confirmed by the biopsy in 58 patients who were all tested positive for HR-HPV DNA. Out of 77 women who had a smear evocative of LGSIL at the first examination with conventional cytology, 56 (72.7%) had a HR-HPV infection. 65 of them had a follow-up and 6 had an underlying HGSIL associated with a HR-HPV. 200 women had a smear evocative of LGSIL with liquid-based cytology with a HR-HPV infection in 168 (84%). In the cohort of 182 women with a follow-up, 8 HGSIL were detected, all associated with HR-HPV.

34 women presented ASCUS with conventional cytology with HR-HPV in 19 of them (55.9%). In 19 women with a follow-up, 5 HGSIL were detected all tested positive for HR-HPV DNA. 175 women presented ASCUS with liquid-based cytology. HR-HPV DNA was detected in 94 cases (53.7%). At the colposcopic control obtained in 135 women, 6 HGSIL were diagnosed all associated with a HR-HPV infection.

In the remaining population of 2145 women with cervical smears within normal limits with conventional cytology, HR-HPV DNA was detected in 231 (10.8%). Out of these 231 women tested HR-HPV positive, according to our protocol, 168 women (72.7%) had a follow-up on a period from 4 to 36 months (median = 24 months). A persistent HR-HPV infection at the second and eventually third examination was observed in 66 women (39.3%) and was associated with the detection of a HGSIL in 15 patients, 4 to 20 months after the first entry (median = 10 months). Out of 5194 women with normal smears with liquid-based cytology, 542 (10.4%) had a HR-HPV infection. In this cohort of 542 women, at the present time, 237 women (43.7%) had a follow-up on a period from 4 to 20 months (median = 12 months). A persistent HR-HPV infection at the second and eventually third examination was observed in 95 women (40.1%) and was associated with the detection of a HGSIL in 10 patients, 4 to 12 months after the first entry (median = 6 months). No HGSIL was detected by cytology and colposcopy in women with regressive HR-HPV infection whatever the initial technique of cytology used. In addition, none of the 1225 women with normal smears and without any HR-HPV infection at the first control (893 with conventional cytology and 332 with liquid-based cytology), developed HGSIL detectable at the second systematic cytological control and at the colposcopic examination performed in 172 of them, on a period of 24 to 36 months (median = 30 months).

### Results according to the technique of cytology and the age of women

Tables 4 and 5 represent the respective sensitivity, specificity, positive and negative predictive values of the various methods used for the detection of a HGSIL histologically proven in the global population and in women aged >30 years. In these tables, only results of cytology and HPV testing at the first examination have been considered for the evaluation of these techniques, since most women had a liquid-based cytology for their follow-up. As expected, liquid-based cytology gave a significantly higher sensitivity (87.8% in the general population and 84.4% in women aged > 30 years) than conventional cytology (68.1% in the general population and 57.7% in women aged > 30 years) ($P < 0.05$). However, the sensitivity of HR-HPV testing remained higher, at 100% in all cases. Nevertheless, if the negative predictive value was always of 100%, the specificity and the positive predictive value of the HR-HPV testing were clearly lower than those of the cytology whatever the technique used. Limiting the screening to women aged > 30 years slightly increased the specificity of the HR-HPV testing to 88.4% in scrapes treated for liquid-based cytology and to 90.1% in smears examined with conventional cytology, but did not modify the positive predictive value for HGSIL detection. However, we have to emphasize that in our same referral population, with the same follow-up protocol, 58 out

**Table 3** Follow-up from the first smear to the final diagnosis

| First smear | | | | First smear | | | |
|---|---|---|---|---|---|---|---|
| Conventional cytology | HR-HPV detection | Follow-up | HG detected at the histology | Thin-prep cytology | HR-HPV detection | Follow-up | HG detected at the histology |
| 2145 WL (94.0%) | 231 (10.8%) | 168 HPV+<br>893 HPV – | 15 HG | 5194 WL (91.9%) | 542 (10.4%) | 237 HPV +<br>332 HPV– | 10 HG |
| 34 ASCUS (1.5%) | 19 (55.9%) | 19 HPV +<br>7 HPV – | 5 HG | 175 ASCUS (3.1%) | 94 (53.7%) | 89 HPV +<br>46 HPV – | 6 HG |
| 77 LG (3.5%) | 56 (72.7%) | 54 HPV +<br>11 HPV – | 6 HG | 200 LG (3.5%) | 168 (84.0%) | 160 HPV +<br>22 HPV – | 8 HG |
| 25 HG (1.1%) | 25 (100.0%) | 25 HPV + | 21 HG | 82 HG (1.4%) | 79 (96.3%) | 79 HPV +<br>3 HPV – | 58 HG |
| 2281 | 331 | 1177 | 47 HG | 5651 | 883 | 968 | 82 HG |

WL: without lesion; LG: low-grade lesion; HG: high-grade lesion; HR-HPV: high-risk HPV.

© 2001 Cancer Research Campaign 

**Table 4** Evaluation of cytology and high-risk HPV testing for the detection of histologically proven high-grade lesion

| Methods | Sensitivity (95% C.I.) | Specificity (95% C.I.) | PPV (95% C.I.) | NPV (95% C.I.) |
|---|---|---|---|---|
| HPV detection | 47/47 (100%)<br>93.8–100% | 1950/2234 (87.3%)<br>85.9–88.7% | 47/331 (14.2%)<br>10.4–18.0% | 1950/1950 (100%)<br>99.8–100% |
| Conventional cytology | 32/47 (68.1%)<br>55.4–79.2% | 2130/2234 (95.3%)<br>94.5–96.2% | 32/136 (23.5%)<br>16.4–30.7% | 2130/2145 (99.3%)<br>99.0–99.6% |
| HPV detection | 82/82 (100%)<br>96.4–100% | 4768/5569 (85.6%)<br>84.7–86.5% | 82/883 (9.3%)<br>7.4–11.2% | 4768/4768 (100%)<br>99.9–100% |
| Thin prep cytology | 72/82 (87.8%)<br>80.3–93.3% | 5184/5569 (93.1%)<br>92.4–93.8% | 72/457 (15.7%)<br>12.4–19.1% | 5184/5194 (99.8%)<br>99.7–99.9% |

PPV: Positive predictive value, NPV: Negative predictive value, C.I.: Confidence interval.

**Table 5** Evaluation of cytology and high-risk HPV testing for the detection of a histologically proven high-grade lesion in women aged > 30 years

| Methods | Sensitivity (95% C.I.) | Specificity (95% C.I.) | PPV (95% C.I.) | NPV (95% C.I.) |
|---|---|---|---|---|
| HPV detection | 26/26 (100%)<br>89.2–100% | 1373/1524 (90.1%)<br>88.6–91.6% | 26/177 (14.7%)<br>9.5–19.9% | 1373/1373 (100%)<br>99.8–100% |
| Conventional cytology | 15/26 (57.7%)<br>39.1–75.0% | 1457/1524 (95.6%)<br>94.6–96.6% | 15/82 (18.3%)<br>9.9–26.6% | 1457/1468 (99.2%)<br>98.5–99.4% |
| HPV detection | 45/45 (100%)<br>93.6–100% | 3603/4076 (88.4%)<br>87.4–89.4% | 45/518 (8.7%)<br>6.3–11.1% | 3603/3603 (100%)<br>99.9–100% |
| Thin prep cytology | 38/45 (84.4%)<br>73.2–92.8% | 3863/4076 (94.8%)<br>94.1–95.5% | 38/251 (15.1%)<br>10.7–19.6% | 3863/3870 (99.8%)<br>99.5–99.8% |

PPV: Positive predictive value, NPV: Negative predictive value, C.I.: Confidence Interval.

**Table 6** Evaluation of the viral load (VL) estimated by the HC-II assay for the detection of a high-grade lesion in cytologically normal smears

| Methods | VL | Sensitivity | Specificity | PPV | NPV |
|---|---|---|---|---|---|
| HPV testing associated with conventional cytology | >3 | 15/15 (100%) | 53/216 (24.5%) | 15/178 (8.4%) | 53/53 (100%) |
| | >10 | 15/15 (100%) | 88/216 (40.7%) | 15/143 (10.5%) | 88/88 (100%) |
| HPV testing associated with thin prep cytology | >3 | 10/10 (100%) | 203/532 (38.1%) | 10/329 (2.9%) | 203/203 (100%) |
| | >10 | 7/10 (70%) | 313/532 (58.8%) | 7/226 (3.1%) | 313/316 (99.0%) |

PPV: Positive predictive value; NPV: Negative predictive value.

**Table 7** Evaluation of cytology and high-risk HPV testing for the detection of a histologically proven high-grade lesion in function of the viral load (VL)

| Methods | Sensitivity (95% C.I.) | Specificity (95% C.I.) | PPV (95% C.I.) | NPV (95% C.I.) |
|---|---|---|---|---|
| HPV detection. VL>3 | 44/47 (93.6%)<br>83.5–98.6% | 2008/2234 (89.9%)<br>88.6–91.1% | 44/270 (16.3%)<br>11.2–20.7% | 2008/2011 (99.8%)<br>99.6–99.7% |
| HPV detection. VL>10 | 42/47 (89.4%)<br>78.4–96.1% | 2049/2234 (91.7%)<br>90.6–92.9% | 42/227 (18.5%)<br>13.5–23.6% | 2049/2054 (99.7%)<br>99.5–99.9% |
| Conventional cytology | 32/47 (68.1%)<br>55.4–79.2% | 2130/2234 (95.3%)<br>94.5–96.2% | 32/136 (23.5%)<br>16.4–30.7% | 2130/2145 (99.3%)<br>99.0–99.6% |
| HPV detection. VL>3 | 80/82 (97.6%)<br>91.9–99.7% | 5007/5569 (89.9%)<br>89.1–90.7% | 80/642 (12.5%)<br>9.9–15.0% | 5007/5009 (99.9%)<br>99.8–99.9% |
| HPV detection. VL>10 | 72/82 (87.8%)<br>80.3–93.3% | 5137/5569 (92.2%)<br>91.6–92.9% | 72/504 (14.3%)<br>11.2–17.3% | 5137/5147 (99.8%)<br>99.7–99.9% |
| Thin prep cytology | 72/82 (87.8%)<br>80.3–93.3% | 5184/5569 (93.1%)<br>92.4–93.8% | 72/457 (15.7%)<br>12.4–19.1% | 5184/5194 (99.8%)<br>99.7–99.9% |

PPV: Positive predictive value; NPV: Negative predictive value; C.I.: Confidence interval.

*© 2001 Cancer Research Campaign*

of 129 HGSIL (45.0%) were detected in women aged < 30 years. This finding explains the absence of modification of the positive predictive value after 30 years.

### Value of the semi-quantitative viral load estimated by the HC-II assay for predicting the persistence of HR-HPV infection and the apparition of HGSIL

Table 6 represents the evaluation of different viral load estimated in RLU (3 and 10 RLU) by the HC-II assay for predicting the detection of HGSIL in smears within normal limits at the first cytological examination with conventional and liquid-based cytology. The sensitivities remained high but the specificities were very low in any case. Table 7 evaluates the efficiency of different viral load values (1,3 and 10 RLU) for general screening. The use of a viral load > 10 increased specificities and predictive positive values and the sensitivity of the HPV testing remained very high, equivalent or superior to that of cytology.

### DISCUSSION

This work using the HC-II assay on a series of 7932 French women attending routine cytologic screening clearly confirms the high prevalence of HR-HPV infection with a peak at 23.6% in the third decade and a progressive decrease after 30 years. In the same way, Herrero et al (2000) using PCR have reported such a high prevalence of HPV infection in women under 25 years in rural Costa Rica. The high percentage of women tested positive in our cohort may partly be due to the technique used with the possibility of cross-hybridization with unknown and/or additional HPV types not included in the HC-II assay probe cocktails (Vernon et al, 2000), which may increase the number of positive results. There is also a significant prevalence and/or persistence of HR-HPV infection in women with HGSIL. HR-HPV DNA has been detected in all the 129 women with a HGSIL histologically confirmed. Thus, in our experience at the present time, whatever the collection protocol used for HPV testing, the sensitivity of HC-II for detecting HGSIL is 100%, but the specificity remains low, at 87.6% when the initial sample has been associated with conventional cytology, and at 85.6% when the initial sample has been treated for liquid-based cytology, with positive predictive values of 14.2% and 9.3% respectively. These results are quite similar to those of previous studies using HPV testing with HC-II for primary screening (Cuzick et al, 1999; Schiffman et al, 2000). Moreover, in our work, the sensitivity of liquid-based cytology is also significantly higher than that of conventional cytology (87.8% vs 68.1%) ($P < 0.05$) as previously reported in the literature when the ThinPrep method has been compared to conventional cytology (Papillo et al, 1998; Sherman et al, 1998; Weintraub and Morabia, 2000). However, if liquid-based cytology alone is a very efficient method, HR-HPV testing combined with cytology significantly increases the detection of HGSIL.

The use of HPV testing for triage test in smears evocative of ASCUS and LGSIL has been recently discussed in large series. Adam et al (1998) have found that screening for HPV DNA with PCR, does not have prognostic value in women reported as having ASCUS or smears evocative of low-grade lesions. The ALTS group (2000) has considered that, because a very high percentage of women with cytological evidence of LGSIL are positive for HPV DNA with the HC-II assay, there is limited potential for HPV testing to direct decisions about the clinical management of these women. Indeed in our series, HR-HPV infection is frequent in this population of women (80.9%). In consequence, its use as a discriminating test for colposcopic referral seems of limited interest, even though all the HGSIL detected at the histological control of cytological LGSIL, were positive for HR-HPV DNA. In another way, in the literature, about 50% of smears with ASCUS are tested positive for HR-HPV DNA and 5 to 10% of ASCUS correspond to an underlying HGSIL associated with HR-HPV infection. Thus, Manos et al (1999) and the ALTS group (Solomon et al, 2001) have proposed that, for women with ASCUS, HPV DNA testing can help to identify those who have underlying HGSIL. Our results are in agreement with this proposition since 5 HGSIL have been detected at the colpo-histological control of ASCUS obtained in 26 women with initial conventional cytology and 6 HGSIL in 135 women with liquid-based cytology, all associated with high-risk HPV infection. If we consider the negative predictive value of the HC-II assay of 100%, HPV testing is a reliable test to select women with ASCUS and HR-HPV infection who had to be referred for colposcopy.

In our series, we have observed that in total, 773 out of 7339 women with smears within normal limits presented a HR-HPV infection. These numerous positive results, particularly in women < 30 years old, are largely responsible for the low specificity and positive predictive value of the HPV testing. Numerous HPV infections are known to regress spontaneously, especially in young women and the mean HPV infection duration is between 8 to 14 months (Ho et al, 1998; Franco et al, 1999). However, Rozendaal et al (1996) have emphasized that the women with normal smears and HR-HPV genotypes are 116 times more at risk of developing HGSIL, in contrast of women without HR-HPV. Moreover, the persistence of HR-HPV infection is significantly associated with progressive disease (Ho et al, 1995; Remmink et al, 1995). Indeed, in our follow-up of 168 (72.7%) of 231 women with smears within normal limits with conventional cytology and with HR-HPV infection, 15 (22.7%) out of 66 women with persistent HR-HPV infection have presented a HGSIL within 4 to 20 months after the first entry. At the present time, we have a shorter follow-up of only 237 (43.7%) out of 542 women with normal smears with liquid-based cytology and HR-HPV infection and the number of 10 HGSIL detected may not exactly reflect the total HGSIL present in this population, but these preliminary data are also very promising. In another way, our more frequent follow-up of women with normal smears and HR-HPV positive, induces a bias of selection which logically results in more numerous diagnoses of HGSIL. However, practically, a systematic intensive follow-up in parallel of women with normal smears and without any HR-HPV infection, is difficult to propose and to obtain. Nevertheless, we underline that no HGSIL has been detected in our cohort of 1225 women with initial HR-HPV negative and normal smears, followed with classic biennal or triennal screening. Thus, according to our protocol of follow-up, the detection of a persistent HR-HPV infection selects a population of women with normal smears at risk for developing a HGSIL.

Considering that HPV infection in younger women often spontaneously regress and that the incidence of cervical cancer in women younger than 30 years is very low, numerous authors (Wright et al, 1998; Cuzick et al, 1999; Stoler, 2000) have recommended the use of HPV testing for older women (>30–35 years old). This could improve the specificity and the positive predictive value of the test. Nevertheless, these propositions do

© 2001 Cancer Research Campaign not consider the frequent early age of the first sexual intercourse as a source of HPV infection with a higher risk for developing a HGSIL before 30 years. In our experience, if we reserve HPV testing in women > 30 years old, the positive predictive value of HPV testing is not modified, but the specificity slightly increases from 87.3% to 90.1% in samples associated with conventional cytology and from 85.6% to 88.4% in samples treated for liquid-based cytology. Again, we have to emphasize that 58 out of our 129 patients (45.0%) with HGSIL were women < 30 years old. This clearly explains the absence of modification of the predictive positive values after 30 years. Consequently, limiting HPV testing in women > 30 years in primary screening will miss the increasing number of HGSIL present under this age.

There may be utility in semiquantitative measurement of high viral load to increase the specificity of HPV testing. Indeed, Cuzick et al (1994) have shown that in women with cytologic abnormalities, HPV type 16 positivity at a high-level detected by a semi-quantitative PCR is strongly related to HGSIL. Ho et al (1995) have also suggested that SIL with a high viral load are more likely to persist than those with a low level of HPV DNA. A recent study using a sensitive real time quantitative PCR assay has clearly demonstrated that cervical carcinoma in situ associated with HPV type 16 occurs mainly in HPV type 16 positive women who had consistently high viral loads long term (Ylitalo et al, 2000). Thus, a high viral load may be considered as a risk factor and preferentially observed in potentially evolutive lesions and in HGSIL. This parameter could be semi-quantitatively evaluated by the relative light unit values (RLU) provided by the HC-II assay. Our present results with HC-II show that when this parameter is basically applied for predicting the apparition of a HGSIL in women without any detectable lesions, the specificity is very low <60% but the sensitivity remains very good. Indeed for a cervical sample/positive control ratio >10, the best results are obtained when HPV testing is performed with conventional cytology, with a sensitivity for predicing a HGSIL of 100%, but with a specificity of 40.7% and a predictive positive value of 10.5%. In another way, when a viral load >10 is considered for general screening, the specificities and predicitive positive values increase and the sensitivity is equivalent or better to that of liquid-based cytology for detecting a HGSIL. However we have to underline that the values of viral load are semi-quantitative global measurements for detection of one or multiple HPV types among the 13 in the kit. Recently, Liaw et al (2001) have reported that HPV type 16 infection is generally associated with an increased risk of subsequent acquisition of other types, but does not affect the persistence of these concomitant infections, regardless of type. Thus, the global high viral load detected by HC-II may be rather confusing, reflecting multiple infections which may regress for many of them. Moreover better viral load measurement has to be validated with a standard denominator of number of epithelial cells collected. All these drawbacks limit at the present time the use of viral load provided by HC-II for predicting HGSIL.

Of particular interest is the negative predictive value of 100% obtained in all our series when HPV testing was used for the screening of HGSIL. Clearly, 1225 women with normal smears and without HR-HPV infection did not develop any detectable HGSIL with a median follow-up of 30 months. Thus, HR-HPV detection appears as a reliable predictive test with possible clinical applications. Cytology will likely continue to be the major screening method for the detection of cervical lesions, but it needs a good technical approach and a high professional skill to be highly sensitive. Considering that the mean time from detectable LGSIL to preclinical invasive cancer is 12–13 years (Gustafson and Adami, 1989), Meijer et al (1998) have proposed that women with cytologically normal smears and a negative HR-HPV test could be rescreened every 8 years. If we subscribe to this screening policy, HPV testing combined with cytology would significantly lower the number of cervical smears and the incidence of colposcopy with a more efficient screening. Another proposition is to begin the primary cervical screening with HPV detection. This policy has been proposed in low-resource settings where HPV DNA testing programmes may be easier to implement than cytologic screening (Kuhn et al, 2000; Schiffman et al, 2000). In our wealthier countries, we can use the same cervical scrape for HPV testing and liquid-based cytology. HR-HPV testing would allow to select positive samples treated in a second time for liquid-based cytology which gives a better sensitivity than conventional cytology and a good specificity. Women with cervical abnormalities in their smears will be immediately recalled for colposcopy. A more intensive cytological screening with HPV positive women with normal smears, with an algorithm of 6 to 12 months, to confirm the persistence of HPV infection and/or the occurrence of SIL. All these propositions of new managements for the cervical screening require extensive and multicentric studies in different countries to be validated in terms of efficiency and costs–benefits. In any case, the introduction of the HPV testing represents a new promising technology for primary cervical screening.

### ACKNOWLEDGEMENTS

This work was supported by a grant from the European Community (L'Europe contre le Cancer), the ARC (Association de Recherche sur le Cancer), the ARERS, the Ligue Contre le Cancer (Comités de la Marne, de l'Aisne, de la Haute-Marne et de l'Aube), the CHU of REIMS and by the LIONS Club of SOISSONS. We thank all the gynaecologists and women who participated in this study.

### REFERENCES

Adam E, Kaufman RH, Berkova Z et al (1998) Is human papillomavirus testing an effective triage method for detection of high-grade (grade 2 or 3) cervical intraepithelial neoplasia? *Am J Obstet Gynecol* **178**: 1235–1244

Bosch FX, Manos MM, Munoz N et al (1995) Prevalence of Human Papillomavirus in Cervical Cancer: a Worldwide Perspective. *J Natl Cancer Inst* **87**: 796–802

Clavel C, Bory JP, Rihet S et al (1998a) Comparative analysis of the human papillomavirus detection by Hybrid Capture assay and routine cytology screening to detect high grade cervical lesions. *Int J Cancer* **75**: 525–528

Clavel C, Masure M, Putaud I et al (1998b) Hybrid Capture II, a new sensitive test for human papillomavirus detection. Comparison with Hybrid Capture I and PCR results in cervical lesions. *J Clin Pathol* **51**: 737–740

Clavel C, Masure M, Bory JP et al (1999) Hybrid Capture II-based human papillomavirus detection, a sensitive test to detect in routine high-grade cervical lesions: a preliminary study on 1518 women. *Br J Cancer* **80**: 1306–1311

Cox JT, Lorincz AT, Schiffman MH et al (1995) Human papillomavirus testing by hybrid capture appears to be useful in triaging women with a cytologic diagnosis of atypical squamous cells of undetermined significance. *Am J Obstet Gynecol* **172**: 946–954

Cuzick J, Terry G, Ho L et al (1994) Human papillomavirus DNA in cervical smears as a predictor of high-grade cervical intraepithelial neoplasia. *Br J Cancer* **69**: 167–171

 *© 2001 Cancer Research Campaign*

Cuzick J, Szarewski A, Terry G et al (1995) Human papillomavirus testing in primary cervical screening. *Lancet* **345**: 1533–1536

Cuzick J, Beverley E, Ho L et al (1999) HPV testing in primary screening of older women. *Br J Cancer* **81**: 554–558

Cuzick J, Sasieni P et al (2000) A systematic review of the role of human papillomavirus (HPV) testing within a cervical screening programme: summary and conclusions. *Br J Cancer* **83**: 561–565

Franco EL, Villa LL, Sobrinho JP et al (1999) Epidemiology of acquisition and clearance of cervical human papillomavirus infection in wommen from a high-risk area for cervical cancer. *J Infect Dis* **180**: 1415–1423

Gaarenstroom KN, Melkert P, Walboomers JMM et al (1994) Human papillomavirus DNA geneotypes: prognostic factors for progression of cervical intraepithelial neoplasia. *Int J Gynecol Cancer* **4**: 73–78

Gustafson L and Adami HO (1989) Natural history of cervical neoplasia: consistent results obtained by an identification technique. *Br J Cancer* **60**: 132–141

Herrero R, Hildisheim A, Bratti C et al (2000) Population-based study of human papillomavirus infection and cervical neoplasia in rural Costa Rica. *J Natl Cancer Inst* **92**: 464–474

Ho GY, Burk RD, Klein s et al (1995) Persistent genital human papillomavirus infection as a risk factor for persistent cervial dysplasia. *J Natl Cancer Inst* **187**: 1365–1371

Ho GYF, Bierman R, Beardsley L et al (1998) Natural history of cervicovaginal papillomavirus infection in young women. *N Engl J Med* 338: 423–428

Kaufman RH and Adam E (1999) Is human papillomavirus testing of value in clinical practice? *Am J Obstet Gynecol* **180**: 1049–1053

Koutsky LA, Holmes KK, Critchlow CW et al (1992) a cohort study of the risk of cervial intraepithelial neoplasia grade 2 or 3 in relation to papillomavirus infection. *N engl J Med* 327: 1272–1278

Kuhn L, Denny L, Pollack A et al (2000) Human papillomavirus DNA testing for cervical cancer screening in low-resource settings. *J Natl Cancer Inst* **92**: 818–825

Liaw KL, Khildesheim A, Burk RD et al (2001) A prospective study of human papillomavirus (HPV) type 16 DNA detection by polymerase chain reaction and its association with acquisition and persistence of other HPV types. *J Infect Dis* **183**: 8–15

Lorincz AT (1996) Hybrid capture method for detection of human papillomavirus DNA in clinical specimens. *Papillomavirus Rep* **7**: 1–5

Lorincz AT, Reid R, Jenson AB et al (1992) Human papillomavirus infection of the cervix: relative risk association of 15 common anogenital types. *Obstet Gynecol* **79**: 328–337

Manos MM, Kinney WK, Hurley LB et al (1999) Identifying women with cervical neoplasia using human papillomavirus testing for equivocal Papanicolaou results. *JAMA* **281**: 1605–1610

Meijer CJLM, Helmerhorst TJM, Rozendaal L et al (1998) HPV typing and testing in gynaecological pathology; has the time come? *Histopathology* **33**: 83–86

Papillo, JL, Zarka MA, St-John TL et al (1998) Evaluation of the ThinPrep Pap test in clinical practice. A seven-month 16,314-case experience in Northern Vermont. *Acta Cytologica* **42**: 203–208

Peyton CL, Schiffman MH, Lorincz AT et al (1998) Comparison of PCR and Hybrid Capture-based human papillomavirus detection systems using multiple cervical specimen collection strategies. *J Clin Microbiol* **36**: 3248–3254

Ratnam S, Franco EL and Ferenczy A (2000) Human papillomavirus testing for primary screening of cervical cancer precursors. *Cancer Epidemiol Biomarkers Prev* **9**: 945–951

Remmink AJ, Walboomers JMM, Helmerhoorst TJM et al (1995) The presence of persistent high-risk HPV genotypes in dysplastic cervical lesions is associated with progressive disease: natural history up to 36 months. *Int J Cancer* **61**: 306–311

Riethmuller D, Gay C, Bertrand X et al (1999) Genital human papillomavirus infection among women recruited for routine cervical cancer screening or for colposcopy determined by Hybrid capture II and Polymerase Chain Reaction. *Diagn Mol Pathol* **8**: 157–164

Rozendaal L, Walboomers JMM, Van Der Linden JC et al (1996) PCR-based high-risk HPV test in cervical cancer screening gives objective risk assessment of women with cytomorphologically normal cervical smears. *Int J Cancer* **68**: 766–769

Schneider A, Zahm DM, Kirchmayr R et al (1996) Screening for cervical intraepithelial neoplasia grade 2/3. *Am J Obstet. Gynecol* **174**: 1534–1541

Schiffman, M, Herrero R, Hildisheim A et al (2000) HPV DNA testing in cervical cancer screening. Results from women in a high-risk province of Costa-Rica. *JAMA* **283**: 87–93

Sherman ME, Mendoza M, Lee KR et al (1998) Performance of liquid-based, thin layer cervical cytology: correlation with reference diagnoses and human papillomavirus testing. *Mod Pathol* **11**: 837–843

Solomon D, Schiffman M, Tarone R (2001) Comparison of three management strategies for patients with atypical squamous cells of undetermined significance: baseline results from a randomized trial. *J Natl Cancer Inst* **93**: 293–299

Stoler M (2000) Advances in cervical screening technology. *Mod Pathol* **13**: 275–284

The Atypical Squamous Cells of Undertermined Significance/Low Grade Squamous Intraepithelial Lesions Triage Study (ALTS) Group (2000) Human Papillomavirus testing for triage of women with cytologic evidence of low-grade squamous intraepithelial lesions: baseline data from a randomized trial. *J Natl Cancer Inst* **92**: 397–402

Vernon SD, Unger ER, Williams D (2000) Comparison of human papillomavirus detection and typing by cycle sequencing, line blotting, and hybrid capture. *J Clin Microbiol* **38**: 651–655

Walboomers JMM, Jacobs MV, Manos MM et al (1999) Human Papillomavirus is a necessary cause of invasive cervical cancer worldwide. *J Pathol* **189**: 12–19

Weintraub J and Morabia A (2000) Efficacy of a liquid-based thin layer method for cervical cancer screening in a population with low incidence of cervical cancer. *Diagn Cytopathol* **22**: 52–59

Wright TC Jr, Lorincz AT, Ferris DG et al (1998) Reflex human papillomavirus deoxyribonucleic acid testing in women with abnormal Papanicolaou smears. *Am J Obstet Gynecol* **178**: 962–966

Ylitalo N, Sorensen P, Josepfsson AM et al (2000) Consistent high viral load of human papillomavirus 16 and risk of cervical carcinoma in situ: a nested-case control study. *Lancet* **355**: 2194–2198

Zur Hausen H (1994) Molecular pathogenesis of cancer of the cervix and its causation by specific human papillomavirus types. *Curr Top Microbiol Immunol* **186**: 131–156

*© 2001 Cancer Research Campaign*
